# Rapid sample preparation and low-resource molecular detection of hepatopancreatic parvoviruses (HPV) by recombinase polymerase amplification lateral flow detection assay in shrimps (*Fenneropenaeus merguiensis*)

**DOI:** 10.1371/journal.pone.0276164

**Published:** 2022-11-09

**Authors:** Nina M. Pollak, Omar Fais, Joanna Kristoffersen, Chontida Phuthaworn, Wayne Knibb, Joanne Macdonald

**Affiliations:** 1 Centre for Bioinnovation, University of the Sunshine Coast, Sippy Downs, Queensland, Australia; 2 School of Science, Technology and Engineering, University of the Sunshine Coast, Sippy Downs, QLD, Australia; Suez Canal University, EGYPT

## Abstract

**Background:**

Viral diseases are a major problem in shrimp aquaculture facilities as these diseases reduce growth rates, which inevitably lead to production and profit losses. Hepatopancreatic parvoviruses (HPV) are common diseases in shrimp that appear to be associated with high or low levels of replication in specific genetic lineages. Selective breeding may result in resistance to HPV and improved body traits such as body weight, meat yield and shrimp colour, facilitating shrimp farming. HPV virus titre is commonly determined by quantitative PCR (qPCR), which is a time-consuming method requiring laboratory equipment unsuitable for field implementation. The aim of this study was to develop a simple, robust, rapid and reliable method to detect HPV in low-resource environments.

**Methods:**

We developed a rapid shrimp HPV test that uses (1) a simple three-step sample preparation protocol, followed by (2) isothermal recombinase polymerase amplification (RPA) and lateral flow strip detection (LFD). Analytical sensitivity testing was performed in a background banana shrimp sample matrix, and retrospective testing of *Fenneropenaeus merguiensis* hepatopancreas tissues (n = 33) with known qPCR viral titres was used to determine diagnostic sensitivity and specificity.

**Results:**

The rapid shrimp HPV test could detect as little as 35 genome-equivalent copies per reaction in homogenized *F*. *merguiensis* banana shrimp. Retrospective testing of stored tissues (n = 33) indicated 100% diagnostic sensitivity (95% confidence interval, CI: 86–100%) and 100% specificity (95% CI: 66–100%) for detection of HPV.

**Conclusion:**

The rapid shrimp HPV test could be completed in only 40 minutes, and required only homogenization pestles, some pipettors, and a small heating block for single temperature incubation at 39°C. Critically, our procedure eliminated the time-consuming purification of nucleic acids from samples and when combined with RPA-LFD offers a user-friendly HPV detection format that can potentially be performed on-site. Our approach represents a major step forward in the development of a simple and sensitive end-point method for quick determination of unfavourable HPV virus numbers in shrimp, and has great potential to advance on-site management of shrimps in aquaculture.

## Introduction

Viral diseases are a major concern in aquaculture, especially in shrimp rearing. The hepatopancreatic parvoviruses (HPV) are common viruses of shrimp and fish comprising *Penaeus monodon* densovirus (*Pmo*DNV), *P*. *merguiensis* densovirus (*Pme*DNV), and *P*. *chinensis* densovirus (*Pch*DNV) [1]. Recently reviewed by Dhar et al. [2], these viruses reduce the survival of larvae in banana shrimp (*F*. *merguiensis*) [3], and can stunt growth at juvenile stages in grow-out ponds for both Japanese tiger prawn (*P*. *japonicas*) [4] and farmed black tiger shrimp (*P*. *monodon*) [5,6]. In particular, Owens et al., reported a positive correlation between HPV levels and incidence of septic tubules in banana shrimp (*F*. *merguiensis*), and inferred that a reduction in virus levels could lead to a 14.5% increase in production [3]. HPV has been detected in the ovary, indicating vertical transmission could also be a concern that needs careful monitoring [7]. Moreover, HPV is frequently identified as a co-infection in shrimp with other viruses [5,7–9], and thus could represent a potential biomarker of viral infection in shrimps.

Management of HPV in aquaculture is difficult, and usually performed through challenge tests based on survival and death ratios, with viral titres determined using pleopods. Viral levels are controlled by choosing broodstock from ponds with lower HPV levels, or by selecting lineages or families known to have lower levels of HPV [10]. The efficacy of management on banana shrimp (*F*. *merguiensis*) was recently analysed, demonstrating that after three years the average pond HPV levels increased over time [10]. Choosing broodstock from lower HPV level ponds may be less efficient than originally predicted, possibly because HPV copy number levels differed between individuals from the same pond.

Aquaculture management verification studies are performed using qPCR, which enables accurate identification of appropriate broodstock. However, qPCR requires time-consuming sample preparation and purification with laboratory-based equipment that is not suitable for field implementation. Molecular methods suitable for field detection are becoming more sophisticated through the implementation of isothermal nucleic acid amplification [11]. Among these, recombinase polymerase amplification (RPA) is a novel isothermal PCR alternative that is fast, highly sensitive, and specific [12]. The technology requires only a single incubation temperature of 39°C, and is thus amenable to operation in low resource environments [13,14]. RPA has allowed efficient detection of *Flavobacterium columnare* causing columnaris affecting numerous freshwater fish [15], and both, *Vibrio parahaemolyticus* strains [16] and *Enterocytozoon hepatopenaei* [17], causing acute hepatopancreatic necrosis disease in shrimp. For detection of viral diseases in shrimp, RPA has demonstrated excellent utility for detection of white spot disease [18], infectious hypodermal and hematopoietic necrosis virus [19], decapod iridescent virus [20], and *P*. *stylirostris* densovirus [21], resulting in clinical sensitivities and specificities as high as 100%, and analytical sensitivity as low as 4 genome copies. However, the biggest impediment for low-resource implementation of RPA is sample preparation, as nucleic acid purification is required prior to testing. Purification is a mulit-step procedure requiring column or magnetic beads to bind nucleic acids, which are reliant on laboratory instruments such as centrifuges or automated robotic instruments. Here we report the development of a rapid protocol for preparing samples that can be performed in low-resource environments. We apply this sample preparation technique for detection of HPV using a combination of RPA and lateral flow detection (LFD), resulting in a rapid shrimp HPV test. We demonstrate detection of HPV in banana shrimp (*F*. *merguiensis)* can be performed using only homogenization pestles, a few pipettors and a heating block, to enable portable testing amenable for field situations.

## Material and methods

### Animal samples

All the specimens were identified as banana shrimp (*F*. *merguiensis*) by morphological keys [22]. Shrimp hepatopancreases were aseptically dissected, tissue kept in Ambion RNA*later* Stabilization Solution (Invitrogen, Carlsbad, USA) and stored at -80°C until used. Samples of farmed banana shrimp were collected at harvest time after 140 days of grow-out [23]. Details on rearing conditions, breeding practices, genotyping, and pedigree construction were described previously [10]. The genotypes, based on DNA microsatellites, were used to assign animals to full-sib groups thus establishing a pedigree [23]. In the present study, samples were selected from 3 full-sib groups previously quantified to have low (10^2^−10^3^, n = 6), medium (10^4^−10^5^, n = 12), and high (10^6^−10^7^, n = 6) HPV copy number [10]. Wildtype banana shrimps were HPV-screened by qPCR and identified as HPV negative (n = 9).

### Standard DNA extraction and qPCR

Genomic DNA was extracted using a DNeasy® Blood & Tissue Kit (Qiagen) according to the manufacturer’s instructions. DNA concentration was measured using a NanoDrop™ 2000 spectrophotometer (Thermo Fisher Scientific) and normalised to a concentration of 40 ng/μL. Extracted DNA was stored at -80°C. HPV titres were quantified by qPCR using the SensiMix HRM kit (Bioline) with a Rotor-Gene 6000 thermal cycler (Corbett Research) as described previously [10,23]. Quantification was achieved using a standard curve from a serially diluted HPV DNA sample that had previously been quantified [10]. Validated primer sequences described by La Fauce *et al*. (2007) were used for qPCR: HPV140 forward, 5’-CTA CTC CAA TGG AAA CTT CTG AGC-3’, and HPV140 reverse, 5’-GTG GCG TTG GAA GGC ACT TC-3’ [24].

### RPA oligonucleotides and synthetic DNA

A rapid, low-resource RPA-LFD test for detection of HPV in shrimps was developed by targeting the structural capsid protein gene of HPV, as this region has previously been used for development of qPCR assays *[10,23]*. By analysing multiple sequence alignments of hepandensovirus sequences from a variety of *Penaeus* and *Fenneropenaeus* sp., highly conserved regions were chosen for primer and probe design suitable for use in an RPA reaction. Primers were designed manually via a multiple sequence alignment (**S1 Fig**) using the free to use GeneDoc software program version 2.7 [25] while adhering to the general rules for RPA primer and probe design provided by TwistDX [26]. Six forward primers, six reverse primers and two probes were designed and tested in various combinations to optimize the RPA test. Primers and probes were synthesized by Integrated DNA Technologies (Coralville, IA, USA): a biotin labelled forward primer (5’ Biotin-GGAAACYTCTGARYCMGGRVYYACCGCCGCRCCSC-3’) and FAM-labelled probe (5’-56-FAM-GGCGYTGGAAKGMAYYKYYDGDRTTTYTHCC-Internal dS spacer-TACCCTGCDGWYTCDCC-C3 spacer-3’) were HPLC purified, and unlabelled reverse primer (5’-GTGTTKBAGRTCRAYDGGYTKRTTGCGGKGGC-3’) was desalted. An RPA test uses the recombinase enzyme to insert biotin and FAM-labelled primers and probes into the double stranded template at a single temperature of 39°C for 25 min, enabling low-resource capture of amplicons using simple LFD of the biotin-FAM dual-labelled amplicon [13]. A positive control DNA template corresponding to position 3928–4404 with 477 bp of *P*. *chinensis* hepandensovirus (Accession number: AY008257; 5740 bp) was synthesized as a double-stranded DNA gBlock® from Integrated DNA technologies (Coralville, IA, USA) and quantitated using the Qubit™ dsDNA HS Assay Kit (Thermo Fisher Scientific, Mulgrave, VIC, AU).

### Rapid shrimp HPV test

**Fig 1:**
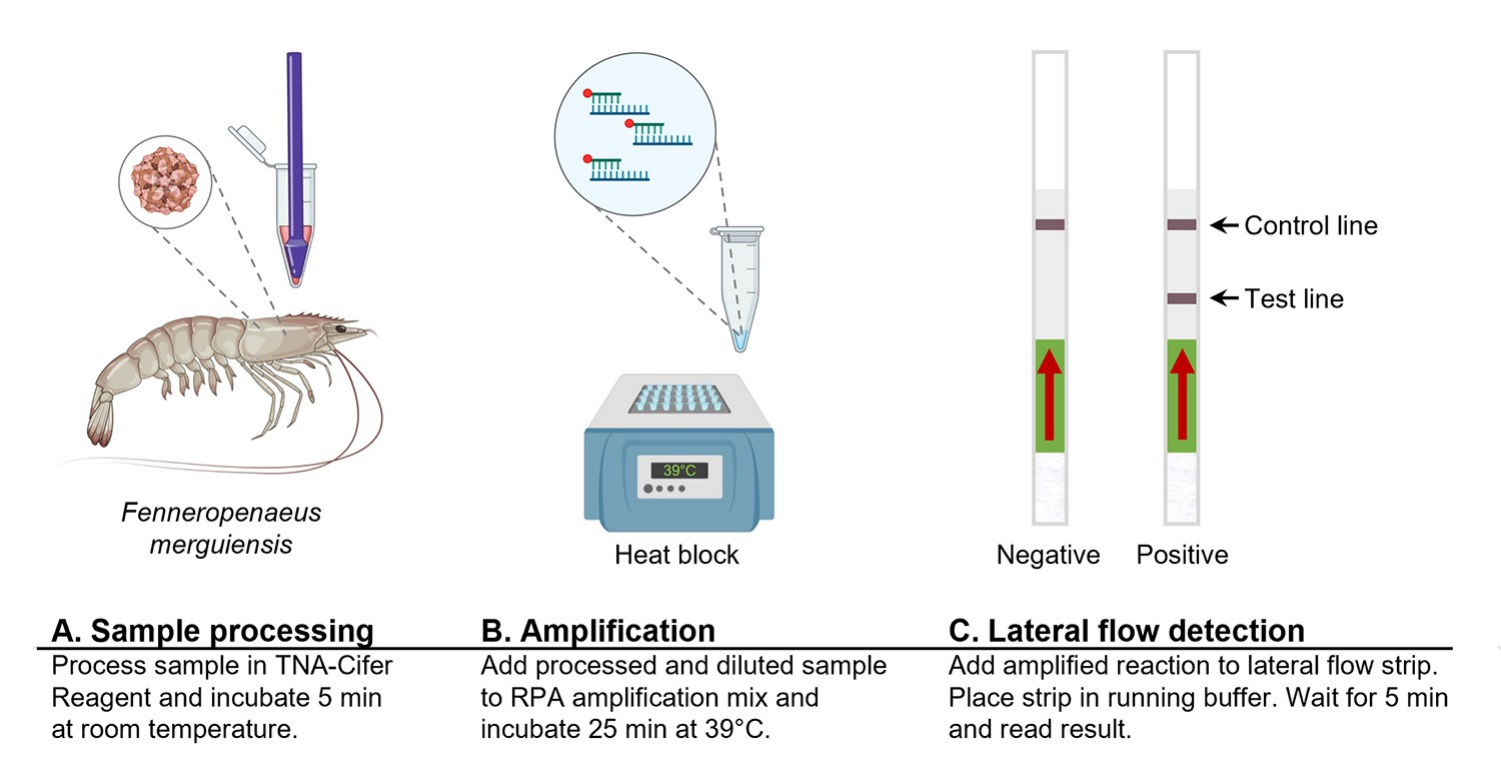
Workflow of rapid shrimp HPV test. Basic workflow of rapid shrimp HPV test including sample processing (**A**), recombinase polymerase amplification (RPA; B), and lateral flow detection (**C**) steps. Created with BioRender.com.

Rapid, field-amenable sample preparation was achieved by collecting ~ 40 μg hepatopancreas tissue and adding 50 μl of TNA-Cifer Reagent (BioCifer, Buderim, Australia) to each 40 μg tissue (resulting in a concentration of 800 ng/μL after processing). The TNA-Cifer Reagent and tissue mix was then homogenized with a pestle in a 1.5 mL tube and incubated for 5 minutes at room temperature. Samples were then diluted 1:5 in nuclease-free water (resulting in a final concentration of 160 ng/μL after dilution) (**Fig 1A**).

RPA tests were performed using a TwistAmp^TM^ nfo Kit (TwistDx, Cambridge UK) as described by the manufacturer with minor adaptions. Briefly, 1x rehydration buffer & 1/5 rehydrated lyophilized pellet, forward primer (420 nM), reverse primer (420 nM), probe (120 nM) and magnesium acetate (14 mM) were mixed with 1 μL diluted sample (~160 ng) or positive control DNA template, to a final reaction volume of 10 μL. The reaction mix was incubated in a heating block for 25 minutes at 39°C, with brief mixing after 10–15 minutes of incubation and at the end of the 25 minutes incubation period (**Fig 1B**). After amplification, 2 μL of RPA amplicons were dropped onto the middle of the sample application area of a preactivated [27] HybriDetect MGHD 1 strip (Milenia Biotec GmbH, Gießen, Germany). Lateral flow dipsticks were then placed into tubes containing 100 μL running buffer (100 mM H_3_BO_3_, 100 mM Na_2_B_4_O_7_, 1% BSA, 0.05% Tween 20, pH 8.8) [28], incubated at room temperature for 5 minutes, results read and imaged immediately (**Fig 1C**). All experiments were performed at least three times if not specified otherwise.

Lateral flow strips were imaged using the MultiDoc-It^TM^ Digital Imaging System (Upland, CA, USA) and analysed using ImageJ software (National Institutes of Health, MD, USA). Greyscale-converted images were used to determine band-intensity, by measuring the mean grey value (limit to threshold), using a fixed area measurement, and subtracting from the maximum mean grey value (255). For each test band, the average of two neighbouring relative white spaces was subtracted from the band intensity to normalize the results. A sample was defined as positive if the normalised band intensity was 3 standard deviations higher than the two neighbouring white space values, representing the cut-off value. Diagnostic test evaluation and comparison used the EpiTools online epidemiological calculator (https://epitools.ausvet.io/comparetwotests) and MedCalc online (https://www.medcalc.org/calc/diagnostic_test.php). Kappa value interpretation is suggested as: values ≤0 as indicating no agreement and 0.01–0.20 as none to slight, 0.21–0.40 as fair, 0.41–0.60 as moderate, 0.61–0.80 as substantial, and 0.81–1.00 as almost perfect agreement.

## Results

### RPA-LFD test analytical sensitivity

**Fig 2:**
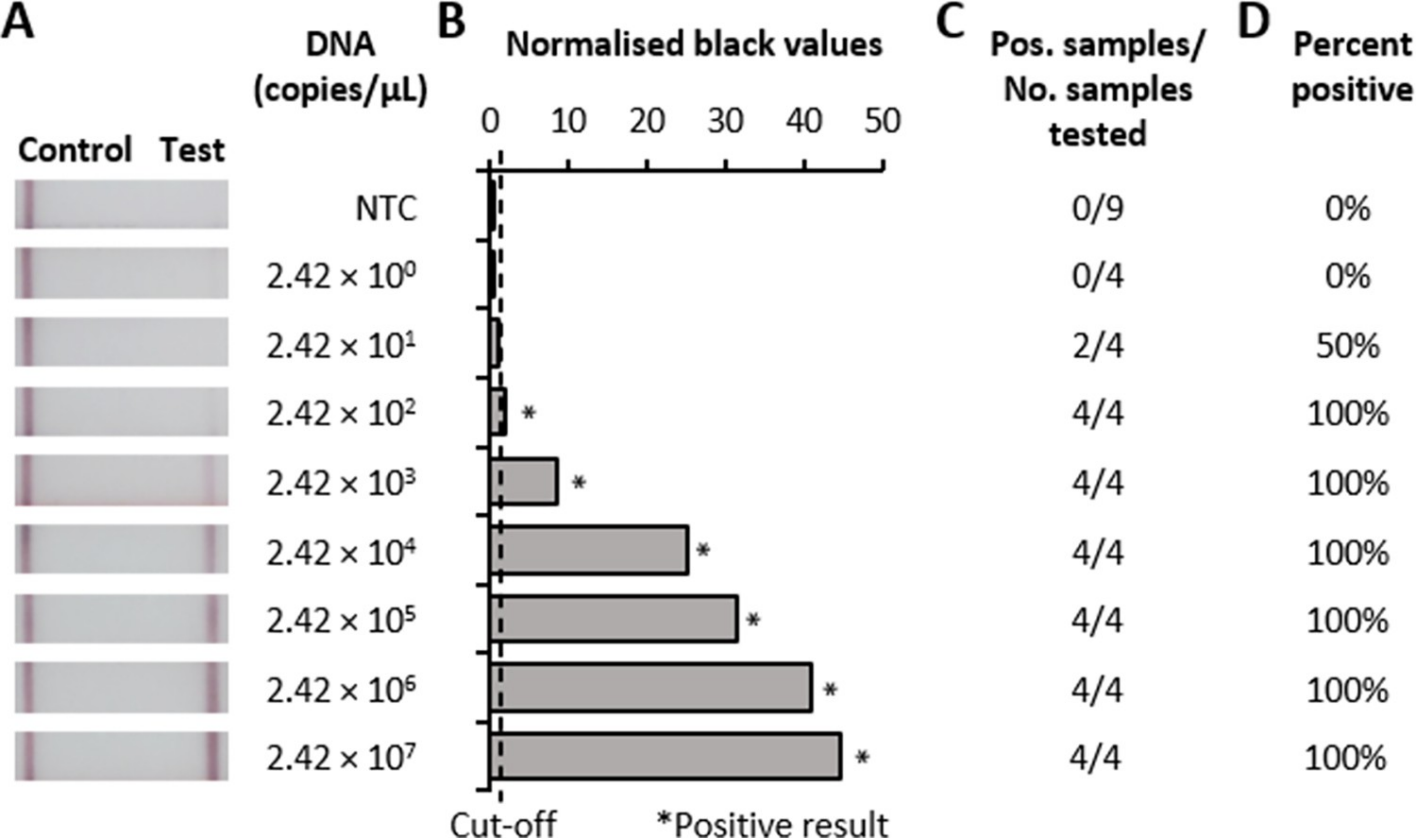
Analytical sensitivity of shrimp HPV RPA-LFD. Sensitivity testing used capsid protein gene fragment plasmid DNA diluted 10-fold in nuclease-free water for Shrimp HPV RPA-LFD (D). **A**, Photograph of lateral-flow strips with control bands (all samples) and test bands (positive samples) compared to copy number of serial diluted synthetic template DNA (copies/μL) and water as negative control (NTC). **B**, Normalised pixel density (normalised black values) from the lateral flow strip displayed in A, which was used to calculate positives (labelled by *) and negatives. **C**, Positive (Pos.) results compared to number (No.) of samples tested at that dilution. **D,** Percentage of positive tests performed at that dilution.

To test the analytical sensitivity of the developed shrimp HPV RPA-LFD test, serial dilutions of a quantified synthetic template DNA were assessed. Strong bands were observed by eye down to 10^4^ copies of DNA, with a fainter positive band visible at 10^3^ copies (**Fig 2**). With digital analysis the HPV RPA-LFD test robustly detected down to <242 genome equivalent copies per reaction. Further, in 2 out of 4 separate runs, the test showed a positive result with 24 copies per reaction. We note that a faint band in the negative controls was observed when running time exceeded 5 minutes, however, the included digital analysis enabled a definitive differentiation between positive and negative results.

### Rapid sample preparation for low-resource detection of HPV-infected banana shrimps

**Fig 3:**
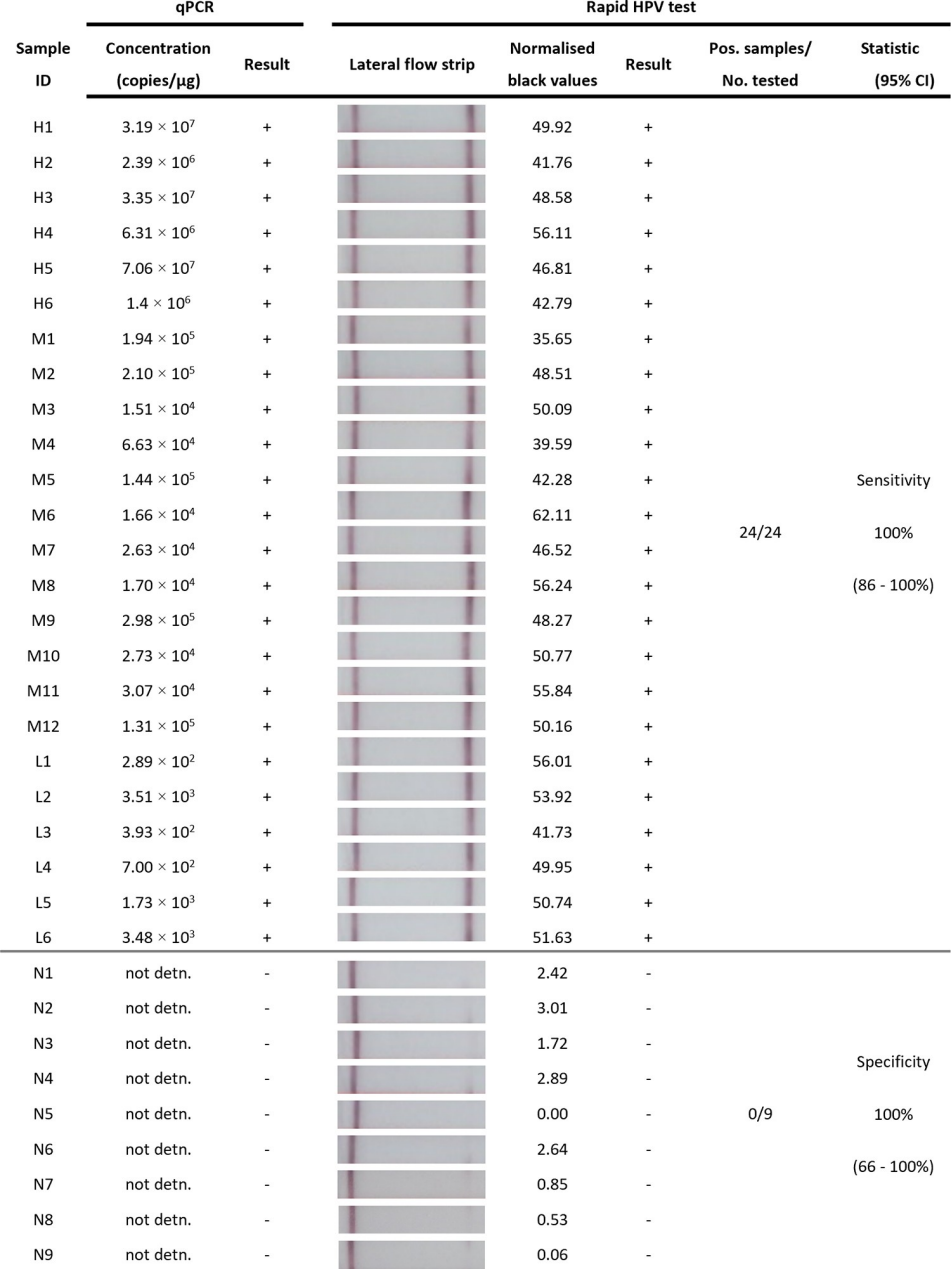
Diagnostic sensitivity and specificity of rapid shrimp HPV test. Each banana shrimp (*F*. *merguiensis*) sample was tested a total of three times in independent test runs showing a representative result. Sample ID: Individual shrimp hepatopancreas with high (H), medium (M), low (L) HPV copy number and uninfected (N) samples; qPCR: concentration (copies/μg) of extracted hepatopancreas sample tested and corresponding test result (+/-); Rapid shrimp HPV test: Image of lateral flow strip result from rapid HPV test (rapid sample processing with TNA-Cifer Reagent followed by shrimp HPV RPA-LFD) [Images of lateral flow strips with two bands (control and test band) indicates the sample is positive for HPV, and single control band indicates a valid reaction with negative sample]; Blank normalised black pixel value from the lateral flow strip with cut-off at 3.96 and corresponding result (+/-); Positive (Pos.) test results compared to total sample number (No.) tested; and Sensitivity and Specificity of the rapid HPV test with 95% confidence interval (CI).

Diagnostic sensitivity testing of stored hepatopancreas tissues (**Fig 3**) indicated our rapid shrimp HPV test consistently detected all HPV-positive samples as HPV-positive (n = 24), regardless of high, medium, or low HPV copy number. In addition, all samples containing non-detectable amounts of HPV by qPCR were also identified by the rapid shrimp HPV test as negative (n = 9). These results (n = 33) indicated the Rapid shrimp HPV test had a high diagnostic accuracy, with 100% diagnostic sensitivity (95% confidence interval, CI: 86–100%) and 100% specificity (95% CI: 66–100%). Further, a Kappa value (95% CI) of 1.00 confirmed rapid HPV test and qPCR results were highly congruent.

### Testing for interfering substances and analytical sensitivity in the shrimp sample environment

**Fig 4:**
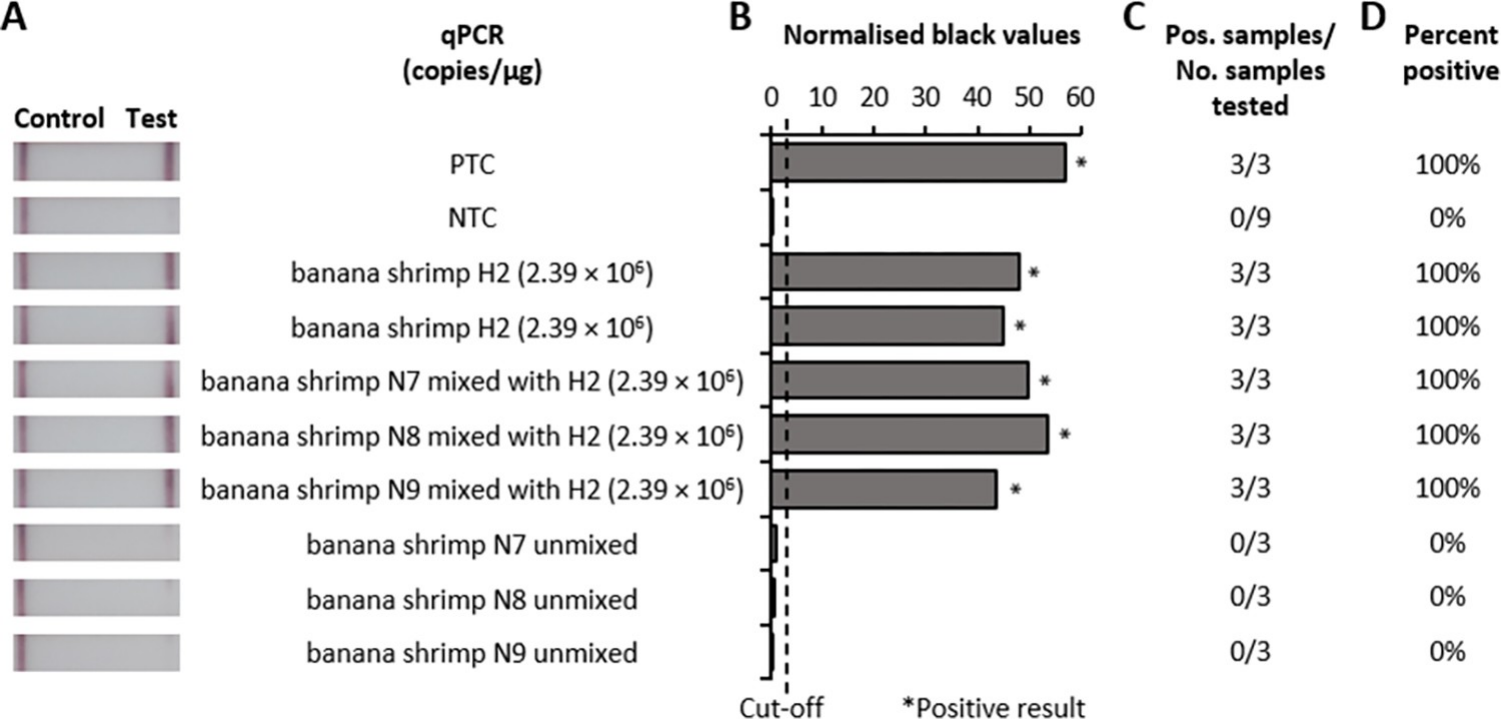
Detection of HPV-positive sample mixed into HPV-negative wildtype banana shrimp samples with the rapid shrimp HPV test. Testing mixed HPV-positive banana shrimp sample (Sample ID: H2, Fig 2) into wildtype samples (Sample IDs: N7-9, Fig 2) at a ratio of 1:1 prior to the required dilution step. Each banana shrimp (*F*. *merguiensis*) sample was tested a total of three times in independent test runs showing a representative result. **A**, Photograph of lateral flow strips from rapid shrimp HPV test (rapid sample processing with TNA-Cifer Reagent followed by HPV RPA-LFD) showing control bands (all samples) and test bands (positive samples) compared to qPCR results (copies/μg), positive control (PTC; synthetic template DNA (2.42 × 10^*6*^ copies/μL)) and water as negative control (NTC). **B**, Normalised pixel density (normalised black values) from the lateral flow strip displayed in A showing positive samples or positive control labelled by *. **C**, Positive (Pos.) test results compared to number (No.) of samples tested in individual runs. **D,** Percentage of positive tests performed for displayed sample.

**Fig 5:**
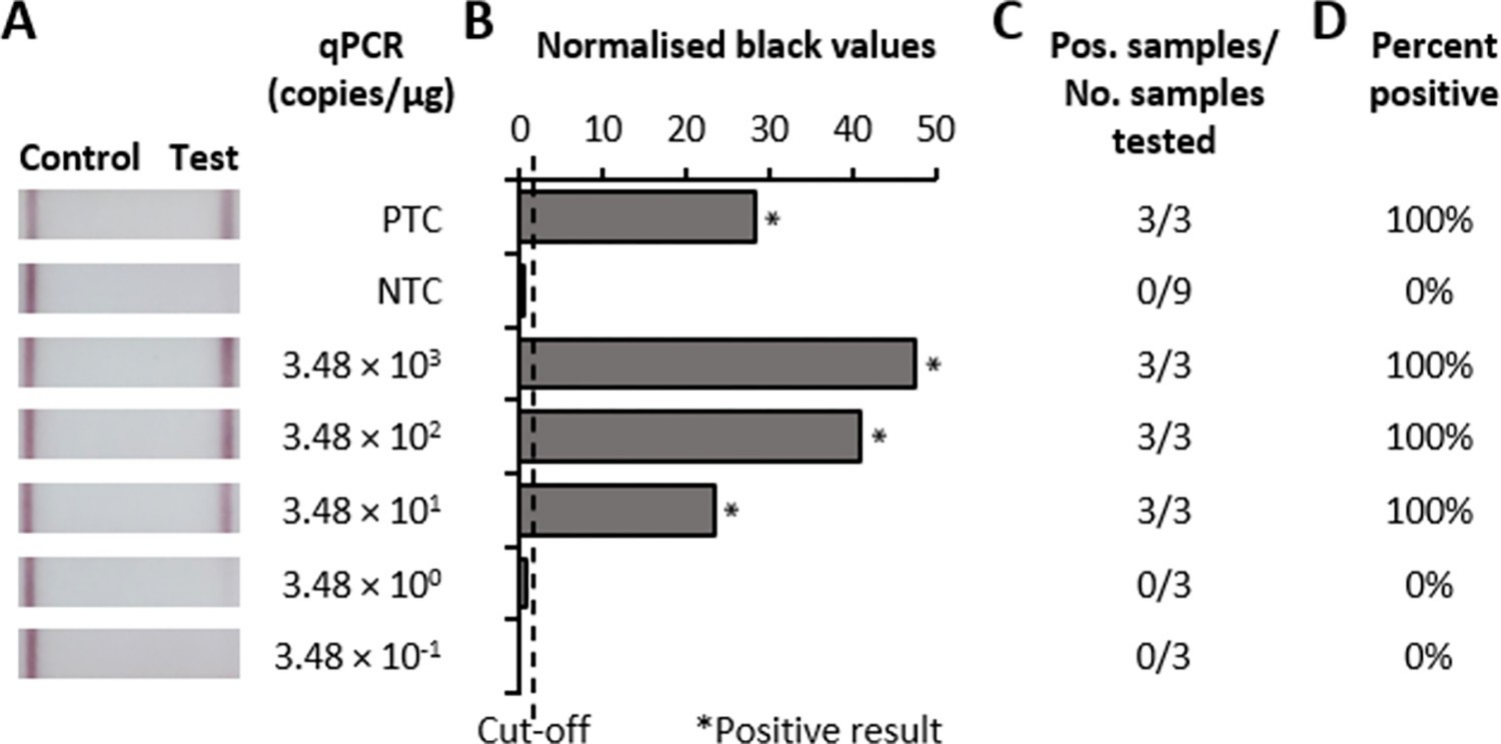
Serial dilutions of a HPV-positive banana shrimp sample assessed with the rapid shrimp HPV test. Testing used 10-fold serial dilutions of a HPV-positive banana shrimp (*F*. *merguiensis*) sample (Sample ID: L6, Fig 2). Dilutions and testing were repeated a total of three times in independent experiments showing a representative result. **A**, Photograph of lateral flow strips with control and test bands compared to qPCR results, water (NTC) or positive control (PTC; 2.42 × 10^*6*^ copies/μL synthetic template DNA). **B**, Normalised pixel density (Normalised black values) from the test displayed in A showing positive samples or positive control (both labelled by *) and negative water control. **C**, Positive (Pos.) test results compared to number (No.) of dilutions tested in individual runs. **D,** Percentage of positive tests performed for displayed sample.

To confirm the rapid shrimp HPV test was not affected by interfering substances, three negative-tested wildtype banana shrimp samples (HPV uninfected determined by qPCR, sample IDs N7-9, **Fig 3**) were processed using our sample preparation method, and mixed 1:1 with a HPV-positive sample (high HPV titre determined by qPCR, sample ID H2, **Fig 3**) that had been similarly processed prior to the required dilution step for RPA-LFD. The test successfully detected HPV in the mixed samples, but not in the unmixed controls (**Fig 4**), demonstrating that the negative results were not due to interfering substances in the samples. We then determined the analytical sensitivity of the rapid shrimp HPV test in the background shrimp sample environment by serially diluting an HPV-positive sample (low HPV titre determined by qPCR, sample ID L6, **Fig 3**) that had been processed using our sample preparation method in negative-tested wildtype banana shrimp samples processed the same way. Results demonstrated the lowest detectable HPV titre was 34.8 genome equivalents, indicating the test could consistently detect <35 genome equivalent copies per reaction (**Fig 5**) in the banana shrimp sample matrix.

## Discussion

Aquaculture management of HPV infections critically requires selection of negative or low-HPV infected broodstock to prevent increasing pond HPV levels over time. Methods to measure HPV levels are difficult, and while qPCR has been demonstrated useful for accruing identifying HPV levels in broodstock, the laboratory and infrastructure requirements of the test precludes implementation for broodstock testing. Here we describe a rapid shrimp HPV test amenable to low-resource implementation, with sensitivity sufficient to detect HPV in stored shrimp hepatopancreas samples with high (10^6^−10^7^), medium (10^4^−10^5^) and low (10^2^−10^3^) copy number of the virus. The test did not detect known qPCR HPV-negative shrimp samples, and this lack of detection was confirmed by spiking positive sample into negative samples to confirm a lack of interfering substances. Retrospective testing of 33 stored hepatopancreas tissues indicated our rapid shrimp HPV test had the requisite diagnostic parameters to ensure accurate estimation of negative shrimp numbers in ponds, with 100% diagnostic sensitivity (95% confidence interval, CI: 86–100%) and 100% specificity (95% CI: 66–100%).

RPA is emerging as an innovative isothermal PCR alternative for rapid detection of diseases. RPA assays are most often developed to enable real-time fluorescence detection, with detection of several viral shrimp diseases described [18–20,29,30] with reported sensitivities ranging from 4 to 100 molecular copies of the pathogens. However, fluorescent reading instruments require additional power requirements and expense that can preclude uptake for low-resource implementation. To date, three tests detecting viruses in shrimp and fish have been developed that couple RPA with the very low-resource amenable detection that uses lateral flow strip detection (LFD) [21,31,32]. These include detection of *Penaeus stylirostris* densovirus [21], cyprinid herpesvirus 2 [31], Micropterus salmoides rhabdovirus [32], with test sensitivities reported detection sensitivities as low as 100 genome copies. These viral RPA-LFD tests [21,31,32] used genomic DNA purification kits to prepare samples, which can be cumbersome to implement in low-resource environments as they require more than 10 steps and laboratory equipment such as centrifuges to perform. Our rapid shrimp HPV test also employed RPA-LFD to enable low-resource detection. However, we developed a rapid three-step sample preparation protocol amenable for application in low-resource settings, reducing sample preparation time to less than 5 minutes. Our procedure only requires a pestle for homogenization and pipettes for liquid handling, which are readily available in low-resource laboratories that implement aquatic diagnostic testing. The improved sample processing for low-resource implementation did not affect the sensitivity of diagnostic detection, with our detection of 35 genome copies per reaction in a banana shrimp sample matrix equivalent to the sensitivities reported using purified nucleic acids.

One of the advantages of the RPA technology in general is the speed of detection. By implementing our rapid sample preparation procedure, our rapid shrimp HPV test had a turnaround time of only 40 minutes, from sample processing to detection. Apart from the simple sample preparation equipment our test required only an additional low-temperature (39°C) heating block. Our entire workflow time is much faster than the 1–2 hour timeframe of qPCR, which requires expensive thermocycling instruments for implementation, and was slightly faster than the 45 minutes incubation at 37°C reported for RPA detection of *Penaeus stylirostris densovirus*, even when the additional sample processing times these other procedures are excluded [21]. Another promising isothermal amplification technology is loop-mediated isothermal amplification (LAMP), and a LAMP test for detection of PmDNV has been previously reported [33] as well as LAMP tests for other shrimp viruses [34–37]. These tests reported sensitivity of detection anywhere from 7–10^8^ copies/reaction, they all required >60 minutes for the LAMP reaction to complete, and additionally required purification of nucleic acids prior to testing. Our rapid shrimp HPV test is faster and simpler to perform than the reported LAMP tests, and even has great potential to replace the current gold standard qPCR, which requires laboratory facilities and experienced personnel.

Limitations of this study include (1) lack of in-field testing for validation to show operational suitability for farmers to screen for HPV, (2) our tests demonstration with dissected hepatopancreas rather than non-invasive testing using faecal matter, (3) lack of analytical specificity testing using other shrimp viruses, and (4) appearance of a faint band in the no template water controls or HPV-negative banana shrimp hepatopancreas samples, if running time of the rapid HPV test lateral flow detection strip exceeded 5 minutes. For the latter point, these faint bands most likely appeared due to sample matrix-induced aggregation of gold nanoparticles or formation of dual-labelled primer dimers, rather than contamination [12,38–40]. Non-specific signals at the test line of lateral flow strips have been associated with batch to batch variation and were previously reduced by preactivating lateral flow strips [27]. However, preactivation of lateral flow strips did not resolve occasional faint test line development for HPV detection. Further optimisation may be achieved by trialling different incubation times and temperatures. To avoid incorrect result interpretation, which potentially generates false positive results, we chose to analyse the lateral flow strips with the freely available software ImageJ. Digital analysis calculating black pixel density allowed unequivocal interpretation of the test. We note, the MultiDoc-ItTM Digital Imaging System could be replaced with a digital camera or a smartphone camera to provide digital analysis in low-resource settings for field implementation.

## Conclusion

In summary, here we describe a rapid and low-resource amenable test for detection of shrimp HPV. The rapid shrimp HPV test described is a sensitive and specific method for rapid detection of HPV within 40 minutes, including sample preparation. This was achieved by developing, for the first time, a simple tissue sample preparation protocol that enabled 5-minute sample preparation, and required only a pestle and some pipettors for liquid handling. The test was able to detect as little as 35 copies of HPV in a *F*. *merguiensis* shrimp sample matrix, and demonstrated 100% diagnostic sensitivity (95% confidence interval, CI: 86–100%) and 100% specificity (95% CI: 66–100%) in retrospective testing of 33 stored *F*. *merguiensis* hepatopancreas tissues with known HPV qPCR titres. Compared to the current gold standard qPCR, the developed test has the advantage of providing a simple, robust, rapid and affordable tool suitable for field-implementation and low-resource environments, which would facilitate on-site management of shrimps in aquaculture.

## Supporting information

S1 Fig Multiple sequence alignment of hepandensoviruses showing regions targeted by the recombinase polymerase amplification (RPA) forward primer, reverse primer, and probe. AY008257.2: *Penaeus chinensis* hepandensovirus; DQ458781: *Penaeus merguiensis* hepandensovirus; EU073937.1: *Penaeus merguiensis* densovirus; EU247528.1: *Penaeus monodon* hepandensovirus; EU346369.1: *Penaeus merguiensis* densovirus; GU371276.1: *Fenneropenaeus chinensis* hepandensovirus; JN082231.1: *Fenneropenaeus chinensis* hepatopancreatic densovirus; NC007218:*Penaeus monodon* hepandensovirus 1; EU290601.1 Hepatopancreatic parvovirus of penaeid shrimp structural protein gene; AF456476.1: Hepatopancreatic parvovirus of penaeid shrimp Thai strain unknown gene; EU588991.1 *Penaeus monodon* hepandensovirus 3 non-structural protein 2 gene; EU617324.1: *Penaeus monodon* hepatopancreatic parvovirus structural protein (HPSP) gene; FJ410797.*2 Penaeus monodon* hepandensovirus 4; JN788268.1 *Penaeus merguiensis* densovirus capsid protein gene; DQ415282.1 Hepatopancreatic parvovirus of penaeid shrimp isolate HPV-3 SDDL nonfunctional capsid protein gene; DQ002873.1 *Penaeus monodon* hepandensovirus 1; AY497195.1 Hepatopancreatic parvovirus of penaeid shrimp strain HPV-4 capsid protein-like gene; AY497193.1 Hepatopancreatic parvovirus of penaeid shrimp strain HPV-2 capsid protein gene; AY497194.1: Hepatopancreatic parvovirus of penaeid shrimp strain HPV-3 capsid protein gene; AY497192.1: Hepatopancreatic parvovirus of penaeid shrimp strain HPV-1 capsid protein-like gene; AY541606.1: Hepatopancreatic parvovirus of penaeid shrimp nonfunctional capsid protein gene; AY945941.1 Hepatopancreatic parvovirus of penaeid shrimp capsid protein gene. * indicates position for internal dS spacer on the probe.
(TIF)
