# Supplementary figures and images for: Rapid sample preparation and low-resource molecular detection of hepatopancreatic parvoviruses (HPV) by recombinase polymerase amplification lateral flow detection assay in shrimps (Fenneropenaeus merguiensis)
Source: PLoS One. 2022 Nov 9;17(11):e0276164. doi: 10.1371/journal.pone.0276164 (PMC9645652; doi:10.1371/journal.pone.0276164)

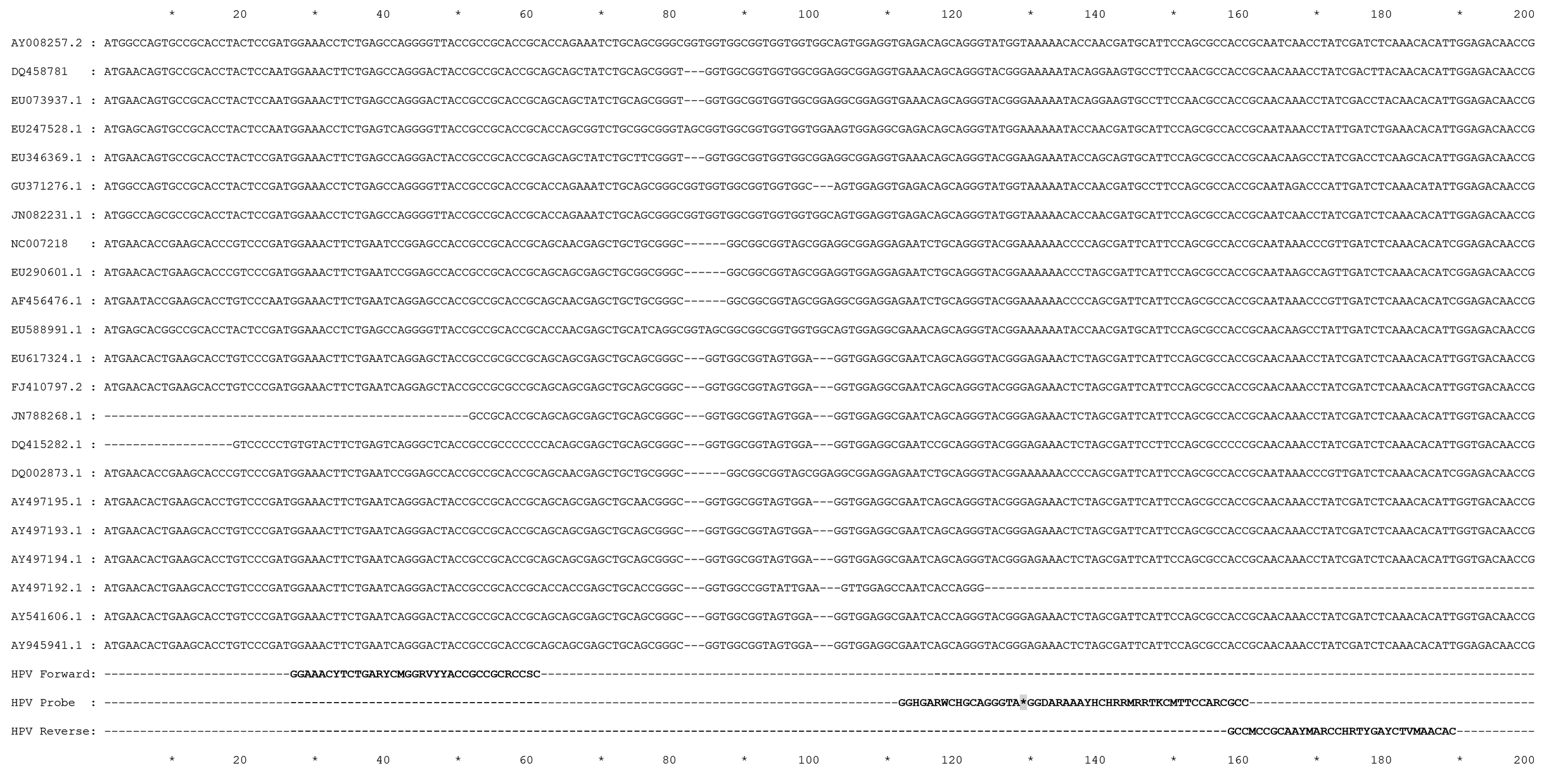

Supplement: S1 Fig — AY008257.2: Penaeus chinensis hepandensovirus; DQ458781: Penaeus merguiensis hepandensovirus; EU073937.1: Penaeus merguiensis densovirus; EU247528.1: Penaeus monodon hepandensovirus; EU346369.1: Penaeus merguiensis densovirus; GU371276.1: Fenneropenaeus chinensis hepandensovirus; JN082231.1: Fenneropenaeus chinensis hepatopancreatic densovirus; NC007218:Penaeus monodon hepandensovirus 1; EU290601.1 Hepatopancreatic parvovirus of penaeid shrimp structural protein gene; AF456476.1: Hepatopancreatic parvovirus of penaeid shrimp Thai strain unknown gene; EU588991.1 Penaeus monodon hepandensovirus 3 non-structural protein 2 gene; EU617324.1: Penaeus monodon hepatopancreatic parvovirus structural protein (HPSP) gene; FJ410797.2 Penaeus monodon hepandensovirus 4; JN788268.1 Penaeus merguiensis densovirus capsid protein gene; DQ415282.1 Hepatopancreatic parvovirus of penaeid shrimp isolate HPV-3 SDDL nonfunctional capsid protein gene; DQ002873.1 Penaeus monodon hepandensovirus 1; AY497195.1 Hepatopancreatic parvovirus of penaeid shrimp strain HPV-4 capsid protein-like gene; AY497193.1 Hepatopancreatic parvovirus of penaeid shrimp strain HPV-2 capsid protein gene; AY497194.1: Hepatopancreatic parvovirus of penaeid shrimp strain HPV-3 capsid protein gene; AY497192.1: Hepatopancreatic parvovirus of penaeid shrimp strain HPV-1 capsid protein-like gene; AY541606.1: Hepatopancreatic parvovirus of penaeid shrimp nonfunctional capsid protein gene; AY945941.1 Hepatopancreatic parvovirus of penaeid shrimp capsid protein gene. * indicates position for internal dS spacer on the probe. (TIF) [file pone.0276164.s001.tif]
